# Responses of cancer cells with wild-type or tyrosine kinase domain-mutated epidermal growth factor receptor (EGFR) to EGFR-targeted therapy are linked to downregulation of hypoxia-inducible factor-1α

**DOI:** 10.1186/1476-4598-6-63

**Published:** 2007-10-11

**Authors:** Yang Lu, Ke Liang, Xinqun Li, Zhen Fan

**Affiliations:** 1 Department of Experimental Therapeutics, The University of Texas M. D. Anderson Cancer Center, Houston, Texas 77030, USA

## Abstract

**Background:**

Searching for novel molecular markers that dependably predict or indicate responses of human cancer cells to epidermal growth factor receptor (EGFR)-targeted therapy is strongly warranted. The purpose of the current study was to evaluate hypoxia-inducible factor-1α (HIF-1α) as a novel response marker compared with previously explored markers following treatment with an EGFR-blocking monoclonal antibody (cetuximab) and a small-molecule EGFR tyrosine kinase inhibitor (gefitinib) in a group of cancer cell lines containing wild-type or tyrosine kinase domain-mutated EGFR.

**Results:**

We found that, compared with previously studied response markers, including EGFR *per se *and three EGFR downstream signal molecules (ERK, Akt, and STAT3), which showed variable post-treatment changes in levels of phosphorylation and no consistent link of the changes to therapeutic responses, HIF-1α showed a selective decrease in protein levels only in responsive cell lines. To demonstrate a critical role of HIF-1α downregulation by EGFR-targeted treatment, we introduced a constitutively expressed HIF-1α mutant (HIF-1α/ΔODD) that is resistant to cetuximab-induced downregulation in a cetuximab-responsive cell line (A431); we found that the HIF-1α/ΔODD-transfected cells remained sensitive to cetuximab-induced inhibition of Akt and ERK phosphorylation but were remarkably less responsive to cetuximab-induced growth inhibition compared with corresponding control cells.

**Conclusion:**

Our data indicates that downregulation of HIF-1α is associated with positive therapeutic responses of cancer cells to EGFR-targeted therapy and suggest further investigation using HIF-1α as an indicator of tumor response to EGFR-targeted therapy in preclinical studies and in the clinical setting.

## Background

Epidermal growth factor receptor (EGFR) has been implicated in the development and progression of a diverse type of solid tumors. Over the past two decades, experimental cancer therapies targeting EGFR have been studied extensively [1-4]. Recent clinical studies have found that targeting EGFR with receptor-blocking monoclonal antibodies such as cetuximab and panitumumab, or with small-molecule EGFR tyrosine kinase inhibitors (TKIs) such as gefitinib and erlotinib, is effective against several types of solid tumors [5-9]. TKI is particularly effective against a subset of non-small cell lung cancers (NSCLCs) that have several somatic mutations in the EGFR tyrosine kinase domain [10-12]. However, many patients do not experience favorable responses to EGFR-targeted therapy, regardless of positive or even high EGFR expression in their tumors [5-9].

Accumulating evidence indicates that the response of cancer cells to EGFR-targeted therapy is a complex process that can be affected by multiple intrinsic and extrinsic resistance mechanisms. Currently, there is a lack of dependable response markers that can objectively predict or indicate therapeutic responses of patients to EGFR-targeted therapies. Exploration of the genetic and biochemical determinants of response to the therapy not only may help identifying patients who would benefit from EGFR-targeted therapy but also may help in the design of co-targeting strategies to improve treatment effectiveness in patients who do not experience an optimal response to EGFR-targeted therapy alone.

We and others recently found that treatment of responsive cancer cells with cetuximab or gefitinib downregulated the levels of hypoxia-inducible factor-1α (HIF-1α) under both normoxic and hypoxic conditions [13,14]. HIF-1α is a component of the HIF-1 heterodimer that is an important transcription factor for the expression of a wide array of genes involved in a variety of cellular functions, including cell cycle traversal, angiogenesis, anti-apoptotic activity, and oxygen homeostasis [15,16]. HIF-1α is overexpressed in a large number of human tumors, and its overexpression correlates with poor prognosis and treatment failure [15,16]. HIF-1α has a very quick turnover rate in normoxia due to an oxygen-dependent ubiquitination and degradation process of the protein [15,16] and is thus constantly replenished by newly synthesized protein in a phosphatidylinositol 3-kinase signaling pathway-dependent manner that may be activated by multiple growth factors or oncogenes [17-22]. This existing knowledge suggests that HIF-1α may be a good indicator of tumor response to EGFR-targeted therapy, but to date no studies have investigated this possibility.

In the present study, we used a group of cancer cell lines with overexpressed EGFR or tyrosine kinase domain-mutated EGFR to determine the association of the cellular responses with response markers to EGFR-targeted therapy with cetuximab and gefitinib. Two recent studies evaluated biochemical changes in cell signaling after cetuximab and gefitinib treatment in association with therapeutic responses of several EGFR wild-type and tyrosine kinase domain-mutated cancer cell lines [23,24]. Amann *et al*. found that both agents induced apoptosis in HCC827 cells (an NSCLC cell line with a 746E-750A in-frame deletion) and that the IC50 (50% inhibitory concentrations) of TKIs and cetuximab were more closely associated with the phosphorylation inhibition of extracellular signaling-related kinase (ERK) and Akt than with EGFR in HCC827, H1819, and H1299 cell lines [23]. Mukohara *et al*. found that gefitinib and cetuximab had similar effects on inhibiting the growth of NSCLC cells with wild-type EGFR (slight inhibition in A549 and H441 cells and moderate inhibition in H1666 cells) but that gefitinib was stronger than cetuximab in inhibiting EGFR-mutated cell lines (H3255, DFCILU-011, and PC-9). In HCC827 cells, both gefitinib and cetuximab induced apoptosis, but gefitinib induced apoptosis to a greater extent than cetuximab [24].

We found in this study that post-treatment downregulation of HIF-1α was more consistently associated with cellular response than were the biochemical changes of ERK and Akt or that of STAT3, another downstream signaling molecule commonly activated by EGFR. When we experimentally elevated HIF-1α expression level by transfecting a constitutively expressed HIF-1α mutant in A431 cells, we found marked resistance of the transfected cells to cetuximab treatment, despite their unchanged sensitivity to cetuximab-induced inhibition of ERK and Akt phosphorylation levels. Our data suggest that HIF-1α is an effective molecular response marker for EGFR-targeted therapy and should be further tested in preclinical studies and in clinical trials.

## Results

### Time- and dose-dependent anti-proliferative and apoptotic responses of cancer cells with wild-type and tyrosine kinase domain-mutated EGFR to cetuximab and gefitinib treatment

**Figure 1:**
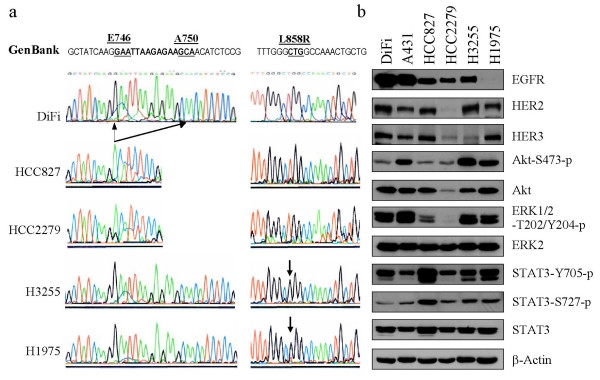
Cancer cell lines with wild-type or tyrosine kinase domain-mutated EGFR. (**a**) PCR fragments from the indicated cell lines were compared with the wild-type sequence of EGFR. The black arrows indicate the codons E746 to A750, which are present in the EGFR in DiFi cells but have been deleted in HCC827 and HCC2279 cells. Codon L858R substitution in H1975 and H3255 cells is indicated by arrows. (**b**) Lysates from the indicated cell lines maintained in regular culture medium were prepared for Western blot analysis using antibodies directed against EGFR, HER2, and HER3, and antibodies directed against total and activation-specific phosphorylated downstream signaling molecules (ERK, Akt, and STAT3). The level of β-actin was used as a reference of lysate protein loading control of each cell line.

Figure 1 shows the genetic and biochemical characteristics of the cell lines used in our study. Compared with the EGFR coding sequences in the GenBank, which originated from A431 cells, no mutations were found in the tyrosine kinase domain of EGFR in DiFi colorectal carcinoma cells (Fig. 1a). In contrast, HCC827 and HCC2279 NSCLC cells had a ΔE746-A750 deletion mutation, and H3255 and H1975 cells had an L858R point mutation. Figure 1b shows the levels of protein expression in the EGFR family, including EGFR (HER1), HER2, and HER3, and the levels of activation-specific phosphorylation of the three most common EGFR substrates, ERK, Akt, and STAT3. At baseline, HCC827, HCC2279, and H3255 cells expressed intermediate levels of EGFR, whereas A431 and DiFi cells expressed high levels. In contrast, H1975 cells expressed the lowest level of EGFR, which was barely detectable unless the film was overexposed. HER2 and HER3 were readily detectable in the cell lines except in H3255 cells (low in HER3) and HCC2279 cells (low in both HER2 and HER3). The basal levels of EGFR downstream signaling, shown by the levels of activation-specific phosphorylation of Akt, ERK, and STAT3, were not consistently associated with the HER family expression levels or EGFR sequence-coding status in a positive or negative manner among the cell lines.

**Figure 2:**
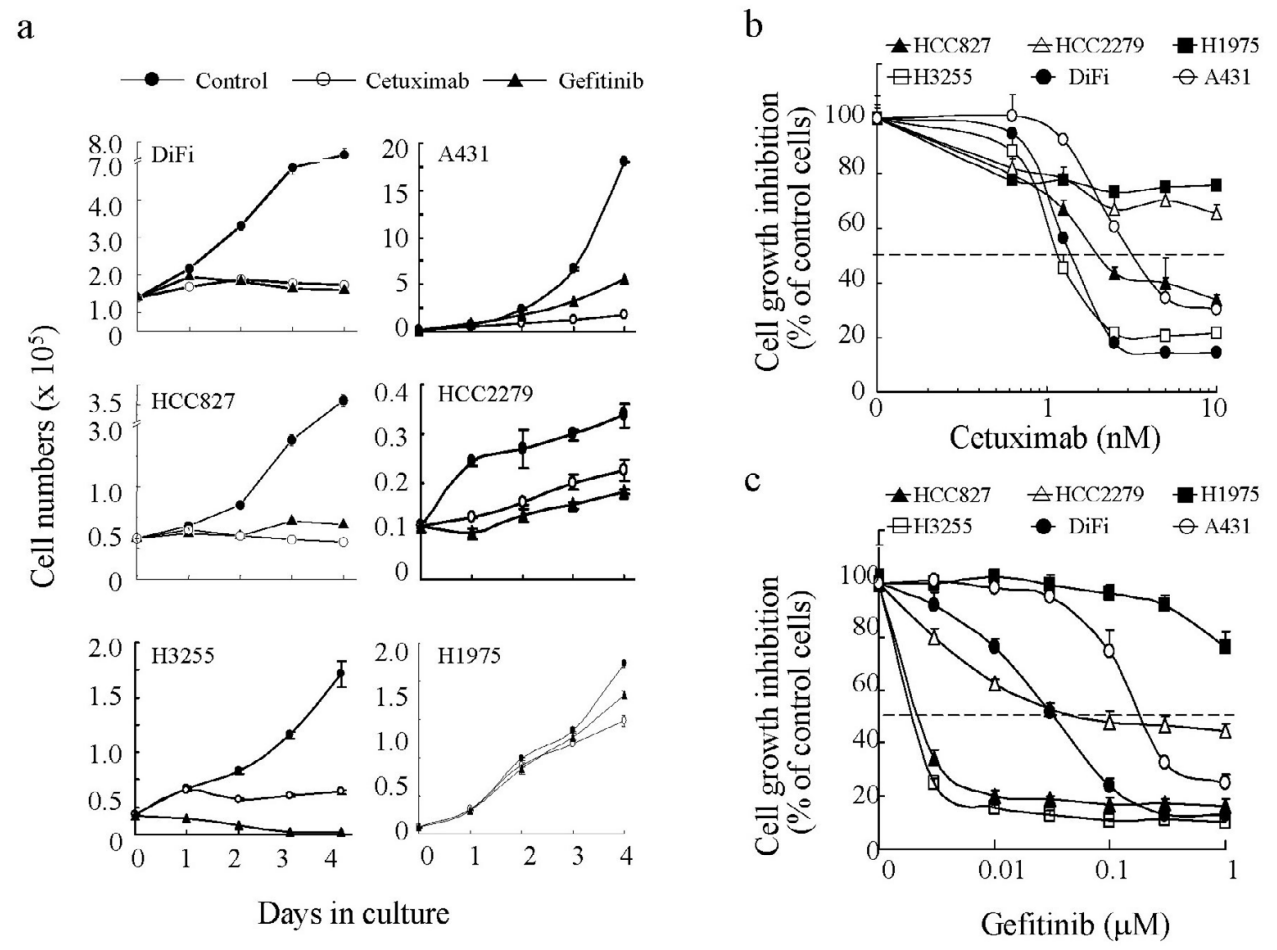
Dose- and time-dependent responses of wild-type EGFR and tyrosine kinase domain-mutated EGFR cells to cetuximab and gefitinib treatment. (**a**) Absolute cell numbers in each treatment group (control [DMSO]), 5 nM cetuximab, and 0.5 μM gefitinib, all in 0.5% FBS medium) were plotted against the duration of treatment. (**b**) The inhibition of cell proliferation after treatment with cetuximab was measured by an MTT assay and is shown as a percentage of the optical density value of control cells (untreated) for each concentration tested. (**c**) The inhibition of cell proliferation after treatment with gefitinib was measured as in (**b**) and is shown as a percentage of the optical density value of vehicle-treated cells (DMSO) for each concentration tested. Results are shown as the mean of five independent measurements, plus or minus the standard deviation (SD). The magnitude of some SDs was smaller than the symbol size; thus some bars do not appear in the figure.

Figure 2a shows the time-dependent responses of these individual cell lines to treatment with 10 nM cetuximab or 0.5 μM gefitinib for 4 days. Four cell lines (DiFi, HCC827, H3255, and A431) showed marked growth inhibition responses after cetuximab or gefitinib treatment, whereas HCC2279 and H1975 cells showed only moderate or poor growth inhibition. The degrees of growth inhibition of DiFi cells after cetuximab (10 nM) and gefitinib (0.5 μM) treatments were comparable, but in A431 and HCC827 cells more growth inhibition was induced by cetuximab than by gefitinib. In contrast, H3255 cells responded more strongly to gefitinib than to cetuximab: massive cell death was microscopically visible just a few hours after exposure to gefitinib, whereas the response of H3255 cells to cetuximab was slower and not evident until those cells had been exposed to the treatment overnight (data not shown). HCC2279 and H1975 cell lines demonstrated much less growth inhibition than did other cell lines in response to treatment with either agent. The growth rate of HCC2279 cells was much slower than that in the other cell lines, which may partly explain the moderate inhibitory effects of the agents on HCC2279 cell proliferation. H1975 cells, which also contain a second mutation (T790M) linked to gefitinib resistance [25], responded poorly to either agent.

Figure 2b shows the simultaneous dose-dependent growth inhibition responses of the cell lines to treatment with serial dilutions of cetuximab for 72 hours. H3255 cells showed a dose-dependent response to cetuximab that was similar to that of DiFi cells, and HCC827 cells showed a response that was less sensitive than that of DiFi cells but better than that of A431 cells at lower doses (~3 nM). The growth of HCC2279 and H1975 cells was minimally inhibited by cetuximab.

In contrast to the results of cetuximab treatment, two EGFR-mutated cell lines, HCC827 and H3255, were far more sensitive to lower (<0.01 μM) concentrations of gefitinib than were DiFi and A431 cells (Fig. 2c). When the gefitinib concentration was ≥ 0.1 μM, DiFi cells exhibited responses comparable to those seen in HCC827 and H3255 cells, but this concentration was still lower than the doses required to produce similar growth inhibition in A431 cells. HCC2279 cells also responded to gefitinib treatment at lower concentrations compared with DiFi and A431 cells, but the maximal level of inhibition was less than that seen in DiFi and A431 cells. Unlike other cells, H1975 cells responded poorly to gefitinib, as reported by other investigators [25].

**Figure 3:**
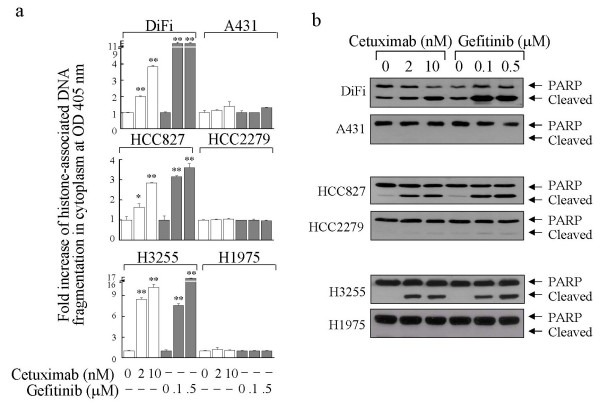
Induction of apoptosis in cancer cells with wild-type EGFR or tyrosine kinase domain-mutated EGFR by cetuximab and gefitinib. Cells from each line were left untreated or were treated with vehicle (DMSO), 5 nM cetuximab, or 0.5 μM gefitinib in a medium containing 0.5% FBS. After 16 hours of treatment, the cells were harvested and lysed for quantitative apoptosis measurement by (**a**) an enzyme-linked immunosorbent assay, as described in the Methods section, and (**b**) Western blot analysis with anti-PARP antibodies. *P < 0.05, **P < 0.01 compared with corresponding controls.

To determine whether cetuximab or gefitinib induced apoptosis in the cell lines, we used two independent apoptosis assays: an enzyme-linked immunosorbent assay to measure the cytoplasmic levels of histone-associated DNA fragments characteristic of apoptotic cells and a Western blot analysis to detect the proteolytic cleavage of poly(adenosine diphosphate-ribose) polymerase (PARP) (Fig. 3a and 3b). Clear evidence of apoptosis was found in three cell lines: DiFi, HCC827, and H3255. At the doses tested, gefitinib seemed to induce a higher cytoplasmic level of histone-associated DNA fragments than did cetuximab (Fig. 3a), but both agents had similar effects on PARP cleavage (Fig. 3b). DiFi cells contained a high basal level of cleaved PARP when cultured in low-serum medium, but the cleaved PARP fragment level clearly increased after treatment. Although cell proliferation was strongly inhibited, no clear sign of apoptosis was detected in A431 cells after treatment with cetuximab or gefitinib under the treatment condition consisting of 0.5% FBS in culture medium. As expected, HCC2279 and H1975 cells showed modest or poor anti-proliferative responses to the treatments, with no apoptosis detected in these cell lines with either treatment.

### Lack of consistency of changes in phosphorylation levels of EGFR and common EGFR substrates in association with positive response to cetuximab and gefitinib treatment

**Figure 4:**
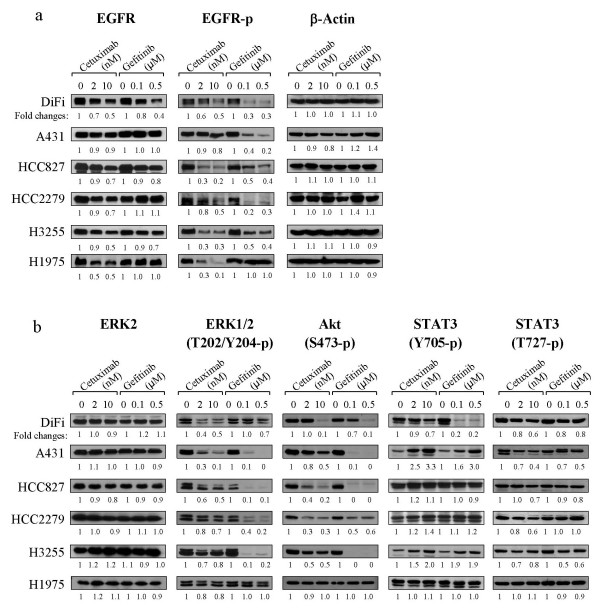
Effects of cetuximab and gefitinib treatment on the levels of total and phosphorylated EGFR and EGFR substrates in cell lines with wild-type and mutated EGFR. Cells from the indicated lines were simultaneously switched to a culture medium containing 0.5% FBS and were either left untreated or treated with cetuximab (2 and 10 nM), vehicle, or gefitinib (0.1 and 0.5 μM) overnight (16 hours). A master medium containing either cetuximab or gefitinib was used in all cell lines. After treatment, the cells were lysed, and equal amounts of cell lysates were subjected to Western blot analysis using antibodies directed against total and phosphorylated EGFR (Y-1068) (**a**) and antibodies directed against phosphorylated ERK, Akt, and STAT3 as indicated (**b**). The levels of β-actin and total ERK served as internal controls for equal protein loading in each lane in (**a**) and (**b**), respectively. The numeric values under each gel were derived from a densitometric analysis of the signals.

Following demonstration of their responses to cetuximab and gefitinib treatment, we next used this group of cell lines to assess the levels of association between growth inhibition response after treatment with cetuximab or gefitinib and the changes in phosphorylation levels of EGFR and common EGFR substrates. Overnight incubation with cetuximab downregulated total EGFR contents by various degrees in all cell lines, except A431 cells (Fig. 4a). The extent of EGFR downregulation was mostly evident in DiFi, H3255, and H1975 cells, indicating that the extent of EGFR downregulation by cetuximab is unrelated to the coding status of the EGFR sequence. Of interest, gefitinib also led to decreased EGFR content in some cell lines (e.g., DiFi and H3255), which was unexpected of a TKI. The degree to which EGFR phosphorylation was inhibited (relative to the controls) also varied. Under comparable experimental doses and treatment conditions, cetuximab was more effective than gefitinib in inhibiting EGFR phosphorylation in HCC827, H3255, and H1975 cells, but gefitinib was more effective than cetuximab in DiFi, A431, and HCC2279 cells. In particular, cetuximab downregulated EGFR levels and inhibited EGFR phosphorylation remarkably more than did gefitinib in H1975 cells; however, cetuximab was only slightly more effective than gefitinib at inhibiting cell proliferation in H1975 cells (Fig. 2), suggesting that the levels of EGFR downregulation or inhibition of phosphorylation after cetuximab and gefitinib treatment are not consistently correlated with positive responses in all cell lines.

ERK1/2, Akt, and STAT3 are three EGFR downstream signal mediators that are commonly evaluated after EGFR-targeted therapy. Figure 4b shows the changes in their levels of activation-specific phosphorylation after treatment of the cells with cetuximab or gefitinib. Despite an overall decrease in ERK1/2 and Akt phosphorylation levels after either treatment in all cell lines except H1975, the degree of ERK1/2 and Akt phosphorylation inhibition varied and could not be quantitatively associated with the extent of cellular responses to the treatment (Figs. 2 and 3). For example, HCC827 cells were far more responsive than HCC2279 cells to cetuximab- or gefitinib-induced growth inhibition and apoptosis, but the patterns of change in their ERK1/2 and Akt phosphorylation levels were similar. Nevertheless, despite the fact that both gefitinib and cetuximab induced apoptosis in DiFi and H3255 cells, gefitinib inhibited ERK1/2 phosphorylation in DiFi cells only modestly compared with cetuximab, but gefitinib inhibited ERK1/2 in H3255 cells far stronger than did cetuximab. Furthermore, gefitinib and cetuximab led to similar levels of Akt phosphorylation inhibition in DiFi cells, but gefitinib was far more effective than cetuximab in H3255 cells. Taken together, these data indicate that the inhibition of ERK and Akt phosphorylation by cetuximab or gefitinib is cell type-dependent.

Even more divergent results were found in the levels of STAT3 phosphorylation after cetuximab and gefitinib treatment: levels of Tyr705-phosphorylated STAT3, which is regulated primarily via the JAK or Src kinase pathway [26], were markedly increased in A431 and H3255 cells; essentially unchanged or modestly increased in HCC827, HCC2279, and H1975 cells; and decreased in DiFi cells. Cell lines with an increased level of Tyr-705-phosphorylated STAT3 after treatment had simultaneous decreases in the levels of Ser-727-phosphorylated STAT3, another important regulatory site that is regulated primarily by ERK1/2 or mTOR kinase [27,28]. In DiFi cells, cetuximab and gefitinib reduced phosphorylation at both sites but to different degrees. In the remaining cell lines, the effects of treatment on Tyr-705 phosphorylated and Ser-727 phosphorylated STAT3 levels were generally similar. An investigation into the possible mechanisms of increased Tyr-705-phosphorylated STAT3 levels in some cell lines is beyond the scope of the current study, but our results indicate that changes in the levels of phosphorylated STAT3 certainly do not consistently reflect cellular responses to cetuximab or gefitinib.

### Downregulation of HIF-1α protein as a positive response marker to cetuximab and gefitinib

**Figure 5:**
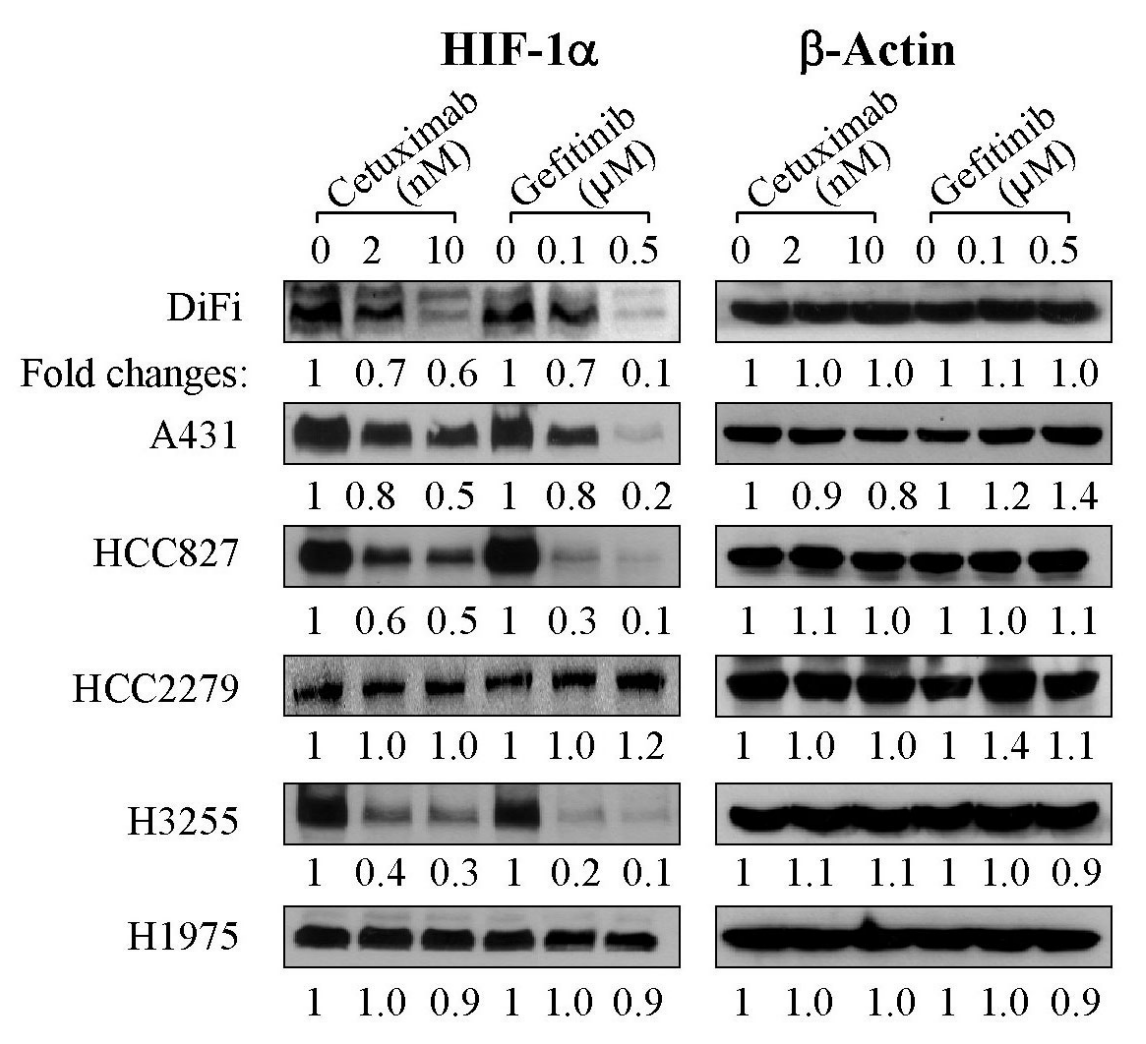
Downregulation of HIF-1α protein levels in cell lines with wild-type or mutated EGFR after treatment with cetuximab or gefitinib. Cells from the indicated cell lines were treated with cetuximab or gefitinib overnight as described in Figure 4. After treatment, the cells were lysed, and equal amounts of cell lysates were subjected to Western blot analysis using antibodies directed against HIF-1α, as indicated. The level of β-actin served as the internal control for equal protein loading in each lane. The numeric values shown under each gel were derived from a densitometric analysis of the signals.

HIF-1α is a well-known transcription factor whose expression is regulated by growth factor- or oncogene-mediated cell signaling via the phosphatidylinositol 3-kinase pathway [15]. Figure 5 shows that, compared with untreated or vehicle-treated cells, the pattern of changes in the levels of HIF-1α protein generally mirrored that in the activation-specific phosphorylation of Akt (Fig. 4b) but seems to be more closely associated with the responses to cetuximab and gefitinib treatment. All four cell lines that responded well to cetuximab or gefitinib treatment (DiFi, A431, HCC827, and H3255) had marked decreases in HIF-1α levels. The patterns of these responses were generally similar among the four cell lines, except that gefitinib induced a higher level of HIF-1α inhibition than did cetuximab. In contrast, in the remaining two cell lines that failed to show an appreciable growth inhibition response to cetuximab and gefitinib treatment, the HIF-1α level did not decrease. These results suggest that HIF-1α is a good indicator of cellular response to EGFR-targeted therapy.

**Figure 6:**
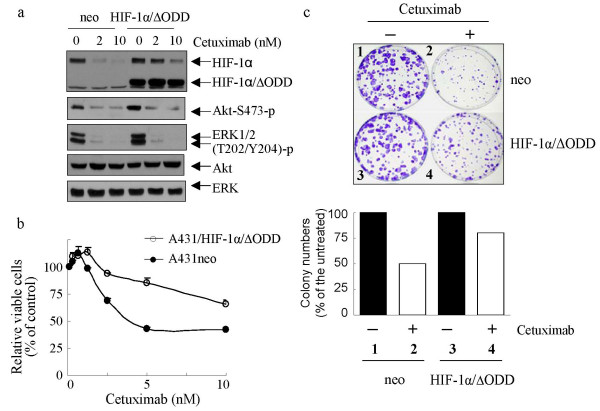
Expression of HIF-1α/ΔODD mutant leads to cellular resistance to cetuximab without affecting cellular sensitivity to cetuximab-induced inhibition of EGFR signaling. (**a**) A431neo and A431/HIF-1α/ΔODD cells were treated as indicated for 16 hours (overnight). Cell lysates were prepared and subjected to Western blot analysis with the indicated antibodies. (**b**) Cells were left untreated or treated with the indicated concentrations of cetuximab in culture with 0.5% FBS for 5 days. Relative cell numbers were measured by the MTT assay and are presented as a percentage of the untreated control. (**c**) A431neo and A431/HIF-1α/ΔODD cells (300 cells/dish) were cultured, with or without cetuximab (2 nM), for 9 days. After treatment, cells were fixed and the colonies were counted, as described in the Methods section.

To provide experimental evidence supporting a critical role of HIF-1α downregulation in mediating cellular responses to EGFR-targeted therapy, we introduced a HIF-1α mutant (HIF-1α/ΔODD) in A431 cells. In the HIF-1α/ΔODD mutant, the oxygen-dependent degradation (ODD) domain of HIF-1α was removed and therefore the mutant became insensitive to VHL ubiquitin ligase-mediated proteasomal degradation, rendering the expressed truncated protein stable in normoxia [29]. After neomycin selection, pooled A431 transfectant cells were obtained and their response to cetuximab was compared with that of control-vector transfected cells. Figure 6a shows that the level of HIF-1α/ΔODD was minimally affected by cetuximab, whereas the level of wild-type HIF-1α was decreased in A431neo and, to a lesser degree, in A431/HIF-1α/ΔODD cells. Importantly, A431/HIF-1α/ΔODD cells remained as sensitive to cetuximab-induced inhibition of ERK and Akt as A431neo cells, as shown by decreased levels of activation-specific phosphorylation in the two molecules. However, the A431/HIF-1α/ΔODD cells were considerably more resistant to cetuximab-induced growth inhibition, as measured by an MTT assay (Fig. 6b). Clonogenic survival assays showed that A431/HIF-1α/ΔODD had markedly more surviving colonies when cultured in the presence of cetuximab than did untreated A431neo cells, indicating that constitutive expression of HIF-1α can indeed render cells resistant to cetuximab treatment (Fig. 6c).

## Discussion

Identification of appropriate markers that dependably mirror the responses of cancer cells to EGFR-targeted therapy is a clinically important undertaking. In this study, we used a group of cancer cell lines with either overexpressed or tyrosine kinase domain-mutated (ΔE746-A750 or L858R) EGFR to assess their responsiveness to cetuximab and gefitinib treatment and to evaluate HIF-1α as novel molecular marker for the therapeutic responses of the cancer cells to EGFR-targeted therapy. We found that the post-treatment decrease in the HIF-1α protein level better correlated with the cellular response than did the decreases in the total or phosphorylated EGFR and phosphorylated EGFR downstream substrate levels (ERK, Akt, and STAT3) in cancer cells with wild-type or tyrosine kinas domain-mutated EGFR. This observation was further confirmed by experimental elevation of the HIF-1α level in the A431 cells, which conferred marked resistance to cetuximab treatment on A431 cells, without affecting cellular sensitivity to cetuximab-induced inhibition of ERK and Akt activation-specific phosphorylation levels.

It is rational to choose HIF-1α as a novel response marker to EGFR-targeted therapy. First, functioning as an inducible binding partner of an important transcription factor regulating many genes involved in tumorigenesis and cancer progression, HIF-1α is an end downstream effector molecule common to the ERK-, Akt-, and STAT3-mediated signal transduction pathways. Because it represents a convergent point of these pathways, HIF-1α is more critical than ERK, Akt, or STAT3 alone in the relay of cell signaling into the nucleus following activation of EGFR [15]. Second, EGFR inhibitors are currently approved and being tested only in solid tumors. Many solid tumors are hypoxic and contain high levels of HIF-1α as a result of increased protein stability; this makes detection of a post-treatment decrease of HIF-1α relatively easy with histological examination of biopsied tumor specimens or by molecular imaging of HIF-1α level changes in the tumors of patients [30,31]. Our data strongly suggest the value of a retrospective review of the correlation of HIF-1α in tumors with responses of the patients to the EGFR-targeted therapies.

In our study, the cellular responses to treatment with cetuximab and gefitinib are consistent with the previous findings of others that mutations in the EGFR tyrosine kinase domain renders the cancer cells sensitive to very low doses of small-molecule TKIs, with the mutations having no effect on cellular sensitivity to monoclonal antibody treatment [24]. However, our data also suggest that the mutation itself is not a sole determinant of apoptosis induction upon EGFR-targeted therapy, because our data show that apoptosis can be induced in cells with either wild-type EGFR (DiFi cells) or the mutated EGFRs (H3255 and HCC827 cells) by either gefitinib or cetuximab treatment. Despite an enhanced sensitivity to low doses of TKI, the maximal therapeutic responses to gefitinib and cetuximab in the EGFR-mutated cells were similar overall when the treatment timing and doses of either agent were appropriate, leading to sufficient inhibition of EGFR kinase activity. Two EGFR-mutated cell lines (HCC827 and H3255) that underwent apoptosis upon gefitinib treatment responded to cetuximab treatment with similar ultimate results (apoptosis), and two other EGFR-mutated cell lines (HCC2279 and H1975) that responded poorly or moderately to gefitinib treatment showed unfavorable responses to cetuximab as well. In particular, H1975 cells contain a second mutation (T790M) reported to be linked to gefitinib resistance [25], but we found that these cells also responded poorly to cetuximab.

It has been proposed that mutations in the tyrosine kinase domain of EGFR cause repositioning of several critical residues surrounding the ATP-binding cleft of the tyrosine kinase domain; this repositioning stabilizes the interactions between the ATP-binding site and reversible inhibitors such as gefitinib [10,11]. Because of their strong interaction, the mutated EGFR can be readily inhibited by TKI with low doses that were usually insufficient to inhibit wild-type EGFR. This speculation is consistent with emerging evidence showing that irreversible EGFR inhibitors decrease the receptor kinase more efficiently than do reversible inhibitors [32]. There is an important caveat, however: although the mutations in the EGFR tyrosine kinase domain result in an enhanced cellular sensitivity to TKI treatment, the mutated EGFR still requires a ligand for receptor activation [10,11,33]. This requirement of ligand binding suggests that EGFR-mutated cells and EGFR wild-type cells should be equally susceptible to cetuximab-mediated receptor blockade. Nevertheless, our data show that cetuximab can indiscriminatingly downregulate EGFR levels in cell lines with either wild-type or mutated EGFR.

Of note, our results on the response of H3255 cells to cetuximab differ from those of another recent study, which reported that H3255 cells were substantially less responsive to cetuximab than to gefitinib [24]. In our study, this was true for only a short time period (6–8 hours) of exposure to cetuximab; cell death was not initially evident in cetuximab-treated cells, whereas gefitinib induced massive cell death that was microscopically visible and measurable via biochemical assays. After the cells had been cultured overnight, however, we found clear evidence of apoptosis in cells treated with cetuximab, both in the level of histone-associated DNA fragments in the cytoplasm and in the cleavage of PARP. By 72 hours, both agents had markedly decreased H3255 cell survival. The time course difference in inducing apoptosis between cetuximab and gefitinib may be explained by the nature of their different working mechanisms: EGFR inhibition induced by cetuximab may require a longer time to effectively block or downregulate EGFR than the time required by gefitinib to shut down kinase activity through competition with ATP binding. In addition, cetuximab is highly specific to EGFR, whereas TKIs are relatively less specific; inhibition of additional targets besides EGFR by gefitinib may also contribute to a faster cellular effect than that of the monoclonal antibody.

## Conclusion

Our data suggest that a decrease in HIF-1α levels is indicative of positive responses to EGFR-targeted therapy of cancer cells with either wild-type or tyrosine kinase domain-mutated EGFR. Genetic aberrations causing an exclusive dependence of cancer cells on the EGFR-mediated cell signaling (i.e., oncogenic addiction), which may be found in cancer cells with either wild-type or tyrosine kinase domain-mutated EGFR, are likely the causes or the molecular determinants of the apoptotic responses of cells to EGFR-targeted therapy. The use of HIF-1α as an indicator of tumor response to EGFR-targeted therapy should be further investigated in preclinical studies and in the clinical setting.

## Methods

### Materials

Cetuximab and gefitinib were gifts from ImClone Systems, Inc. (New York, NY, USA) and AstraZeneca (Wilmington, DE, USA), respectively. All other materials were purchased from Sigma-Aldrich (St. Louis, MO, USA) unless otherwise specified.

### Cell lines and culture

A431 human vulvar squamous carcinoma cells and DiFi colorectal adenocarcinoma cells were described previously [34-37]. HCC827, HCC2279, H3255, and H1975 human NSCLC cell lines were kindly provided by Dr. John Minna of The University of Texas Southwestern Medical Center (Dallas, TX, USA) through Dr. Jonathan M. Kurie of The University of Texas M. D. Anderson Cancer Center (Houston, TX, USA). All cell lines were grown and maintained in Dulbecco's modified Eagle's medium or Ham's F12 medium supplemented with 10% fetal bovine serum (FBS), 2 mM glutamine, 100 U/mL penicillin, and 100 μg/mL streptomycin and incubated in a humidified atmosphere (95% air and 5% CO_2_) at 37°C.

### RNA extraction, cDNA synthesis, polymerase chain reaction, and EGFR sequencing

Total RNA was extracted from the cell lines using a modified chloroform/phenol procedure (Trizol; Invitrogen-Life Technologies, Gaithersburg, MD, USA). First-strand cDNA of the intracellular domain of EGFR was generated using reverse transcriptase (Roche Diagnostics Corp., Indianapolis, IN, USA) and amplified by polymerase chain reaction (PCR) using the Expand high-fidelity PCR system (Roche Diagnostics) with the following primer pair: forward (2137–2157 bp), 5'-AAAAAGATCAAAGTGCTGGGC-3'; and reverse (3643–3625 bp), 5'-CCTCCGTGGTCATGCTCC-3'. PCR products were purified by precipitation with alcohol, and sequences were analyzed by an automated DNA sequence analyzer using the same primers.

### Western blot analysis and blotting antibodies

Cultured cells were harvested with a rubber scraper and washed twice with cold phosphate-buffered saline. Cell pellets were lysed and kept on ice for at least 10 minutes with a buffer containing 50 mM Tris (pH 7.4), 150 mM NaCl, 0.5% Nonidet P-40, 50 mM NaF, 1 mM Na_3_VO_4_, 1 mM phenylmethylsulfonylfluoride, 25 μg/mL leupeptin, and 25 μg/mL aprotinin. The lysates were cleared by centrifugation, and the supernatants were collected. Equal amounts of lysate protein were separated by sodium dodecyl sulfate-polyacrylamide gel electrophoresis, and Western blot analyses were performed with various specific primary antibodies. The antibodies directed against total and Y1068-phosphorylated EGFR, HER2, total and S473-phosphorylated Akt, T202/Y204-phosphorylated ERK, and PARP were obtained from Cell Signaling Technology, Inc. (Beverly, MA, USA). Antibodies directed against ERK and HER3 were obtained from Santa Cruz Biotechnology, Inc. (Santa Cruz, CA, USA). Specific signals were visualized using an enhanced chemiluminescence detection kit (Amersham, Arlington Heights, IL, USA).

### Cell proliferation and survival assays

Time-dependent cell responses to treatments were determined by counting cells. After the various treatments, cells were harvested by trypsinization and counted in a Coulter counter. Dose-dependent cell responses to treatment were determined by MTT [3-(4,5-dimethylthiazol-2-yl)-2, 5-diphenyltetrazolium bromide] colorimetric assays. After treatment, cells were incubated for 2 hours at 37°C in a CO_2 _incubator with 10 mg/mL MTT (50 μL/well). The cells were then lysed with a lysis buffer (500 μL/well) containing 20% sodium dodecyl sulfate in dimethyl formamide/H_2_O (1:1, v/v) (pH 4.7) at 37°C for at least 6 hours. The relative survival of untreated and treated cells was determined by measuring the optical density of cell lysates at a wavelength of 570 nm. Cell viability was expressed as a percentage and calculated as the optical density of the treated cells relative to that of the corresponding control or untreated cells.

### Apoptosis assays

Apoptosis was measured by detecting proteolytic cleavage of PARP using Western blot analysis and by quantifying cytoplasmic levels of histone-associated DNA fragments (mononucleosomes and oligonucleosomes) using an enzyme-linked immunosorbent assay kit (Roche Diagnostics), as we previously reported [38,39].

### HIF-1α construct and transfection

The pcDNA3 expression construct containing the HIF-1α/ΔODD mutant was kindly provided by Dr. L. Eric Huang (University of Utah School of Medicine, Salt Lake City, UT, USA). Transient transfection of the construct was performed with lipofectamine 2000 (Invitrogen, Carlsbad, CA, USA), following the instructions provided by the manufacturer (Roche Diagnostics).

### Monolayer clonogenic assays

Exponentially growing cells were collected from monolayer culture by trypsinization and plated at a low density. Cells were allowed to adhere overnight prior to the addition of 2 nM cetuximab in 10% FBS culture medium for 9 days. Colonies were fixed in 1% crystal violet blue (w/v) in 100% methanol for 30 minutes. Colonies estimated to be larger than 50 cells were counted, and survival was calculated relative to the number of untreated controls.

## Abbreviations

**EGFR**, epidermal growth factor receptor; **HER**, human EGF receptor family; **HIF-1α**, hypoxia-inducible growth factor-1α; **ΔODD**, oxygen-dependent domain deletion mutant of HIF-1α; **ERK**, extracellular signaling-related kinase; **TKI**, small-molecule tyrosine kinase inhibitors; **NSCLC**, non-small cell lung cancers.

## Competing interests

The author(s) declare that they have no competing interests.

## Authors' contributions

YL, KL, and XL performed experiments and interpreted data; the authors' contributions to this research are reflected in the order shown. ZF supervised all aspects of this research and prepared the manuscript. All authors read and approved the final manuscript.
